# Endovascular Graft Infection by *Neisseria meningitidis* : A Rare but Fatal Infection

**DOI:** 10.1055/s-0040-1715088

**Published:** 2020-12-23

**Authors:** Ana Lopez-Marco, Satya Das, Robert Serafino-Wani, Sadasivam Selvakumar, Benjamin Adams, Aung Oo

**Affiliations:** 1 Department of Cardiac Surgery, Barts Heart Centre, St Bartholomew's Hospital, London, United Kingdom; 2 Department of Microbiology, St. Bartholomew's Hospital, London, United Kingdom; 3 Department of Vascular Surgery, Lister Hospital, Stevenage, United Kingdom

**Keywords:** infrarenal aneurysm, EVAR, infection, thoracoabdominal, meningococcal disease

## Abstract

Endovascular graft infection is a rare but challenging complication that requires a tailored approach to remove the infected graft and restore the arterial circulation combined with long-term antibiotic therapy. We present a case surgically treated with explant of the graft and reconstruction of the thoracoabdominal aorta. Microbiological investigation revealed growth of *Neisseria meningitidis* , which is extremely rare in this location, and to our knowledge, has not been previously published in the literature.

## Introduction

Infection of endovascular prosthesis is a rare but challenging complication that requires a tailored approach to remove the infected graft and restore the arterial circulation, combined with long-term antibiotic therapy. Despite surgical repair, morbidity and mortality remain high. 1 2 3

## Case Presentation

We present a 69-year-old male who underwent endovascular aneurysm repair (EVAR) of his infrarenal aortic aneurysm with a modular graft (aortic bifurcated body and two iliac limbs, TriVascular Ovation Prime, and Ovation iX Iliac, TriVascular Inc., Santa Rosa, CA).

**Fig. 1:**
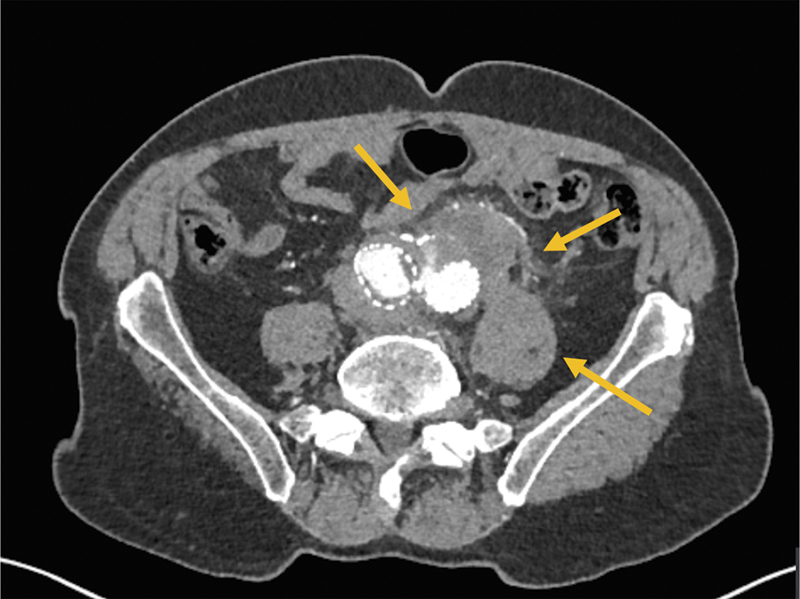
Axial view of the preoperative contrasted computed tomography of the aorta demonstrating a Type-II endoleak and a periaortic gas collection (highlighted by yellow arrows).

**Fig. 2:**
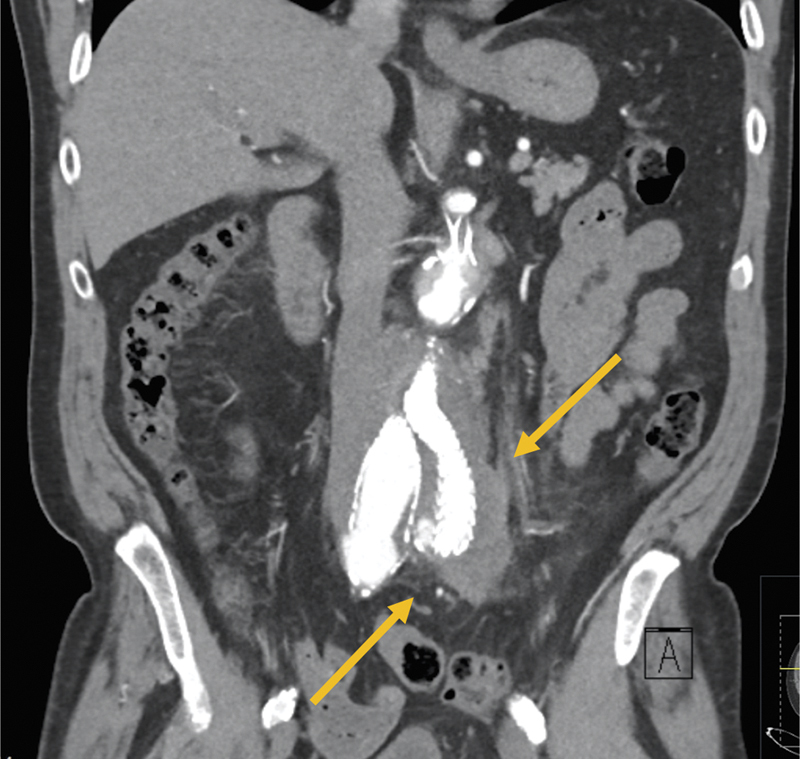
Coronal view of the preoperative contrasted computed tomography of the aorta, demonstrating the presence of a periaortic gas collection extending into the left iliac fossa (highlighted by yellow arrows).

After 18 months, he developed flu-like symptoms with persistent elevated systemic C-reactive protein (CRP; >120 mg/L). Blood cultures were negative. Amoxicillin/clavulanic acid and steroids controlled his symptoms; however, computed tomography (CT) revealed a new fluid and gaseous perigraft collection extending into the left iliac fossa confirming the diagnosis of infected EVAR ( Figs. 1 and 2 ).

A multidisciplinary team (MDT) decided that the best approach was open surgery to remove the infected prosthesis and primarily repair the thoracoabdominal aorta (Crawford's level-IV extent).

**Fig. 3:**
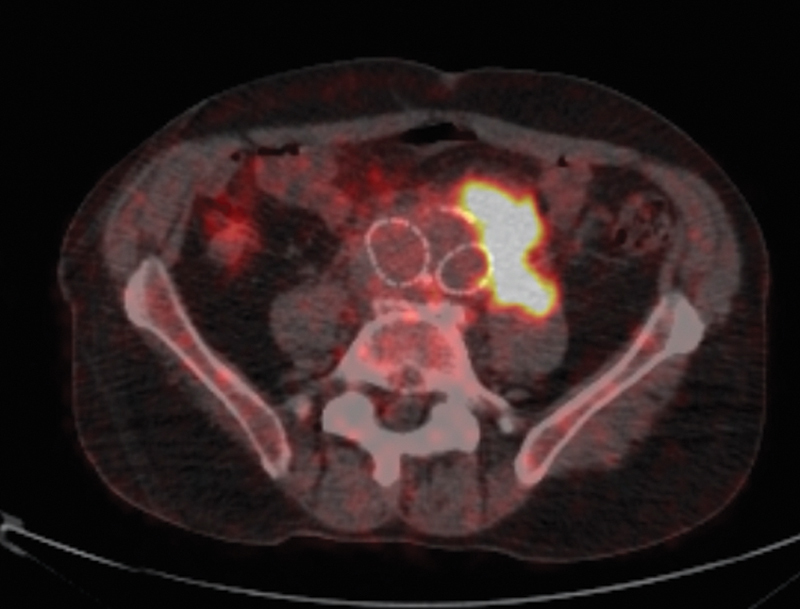
Axial view of the positron emission tomography-computed tomography showing a high uptake extending into the left iliac fossa.

Preoperative workup consisted of a transthoracic echocardiogram that revealed good biventricular function; a CT coronary angiogram that did not identify significant coronary stenosis and a positron emission tomography-CT (PET-CT) that confirmed intense avidity in the infrarenal periaortic tissues ( Fig. 3 ).

He was then prepared for urgent aortic surgery. Intraoperative monitoring consisted of right radial and right femoral arterial lines, venous central line, near-infrared spectrometry for cerebral, paraspinal, and lower limb saturations, and motor-evoked potentials. A spinal drainage system was inserted for spinal protection.

A left thoracophrenolaparotomy was performed through the seventh left intercostal space toward the median abdominal line. The diaphragm was divided circumferentially and the abdominal aorta approached via retroperitoneal space.

The descending thoracic aorta (DTA) was mobilized, leaving the visceral segment undisturbed to avoid complications.

The left inferior pulmonary vein was cannulated with a 28-F cannula in preparation for left heart bypass. The left femoral artery was not used due to extensive calcification.

The distal DTA was clamped, and the aorta was opened longitudinally along the visceral segment; distal perfusion to the limbs was established via two separate 13-F balloon-tipped cannula placed into the iliacs.

**Fig. 4:**
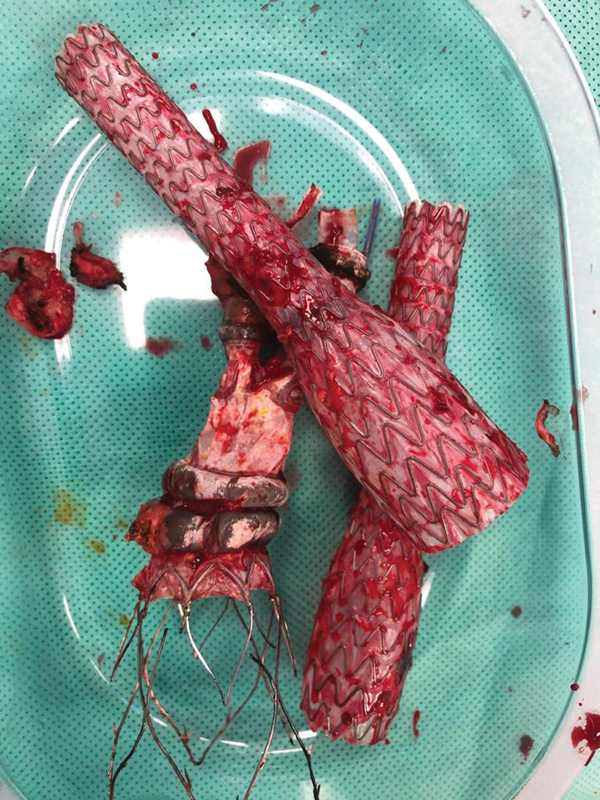
Modular components of the prosthesis used for the endovascular aneurysm repair after the surgical explantation.

Removal of the main body of the EVAR ( Fig. 4 ) was then performed and visceral protection established by administering cold blood through the celiac axis and the superior mesenteric artery (SMA) with two separate 13-F balloon-tipped cannulae and 1 L of Custodiol solution with two separate 10-F balloon-tipped cannula into the renal arteries.

A 20-mm Rifampicin-soaked four-branched graft was anastomosed to the distal DTA at the level of the diaphragm with a two-layered buttressed 3/0 Prolene.

Reimplantation of the visceral vessels was performed with continuous 4/0 Prolene in the following sequence: celiac axis, SMA, right renal, and left renal.

The remaining modules of the EVAR were then fully removed ( Fig. 4 ), as well as the inflammatory tissue and clots within the aneurysmal sac and the left iliac fossa abscess.

A 30-mm straight Dacron graft was anastomosed to the suprailiac aortic segment with the same buttressed technique used for the proximal anastomosis and finally, the two Dacron grafts were anastomosed together.

When the infrarenal aorta mobilization started, the patient showed signs of systemic inflammatory response and sepsis (SIRS), requiring increasing fluid replacement therapy that generated a shift of fluids to the third space and progressive swelling of the intraperitoneal contents. The thoracolaparotomy wound was left open with the cavity packed with surgical swabs.

Postoperative period was marked by profound hypotension and vasodilation secondary to SIRS. The patient developed progressive multiorgan failure and died in the early morning hours.

Cultures of the infected graft and evacuated pus collection revealed a profuse growth of *Neisseria meningitidis* from three different samples.

## Discussion

Graft infections after endovascular abdominal aneurysm repair are rare (incidence < 1%); however, they are extremely challenging to manage, are associated with significant morbidity and mortality, and are potentially lethal if untreated. 1 2 3

The foreign material of the graft and the thrombus within the aneurysm sac provide a nidus where microorganisms can grow. Predominant microorganisms are gram positive ( *Staphylococci* and *Streptococci* ), although gram-negative enteric organisms and polymicrobial infections are not rare. 1 2 3 4

Diagnosis is complex and requires a high index of suspicion, based on a combination of clinical symptoms, imaging and microbiological investigations (negative cultures in up to 33% of the cases). 1 2 3 4

Angio-CT is the gold-standard imaging technique, looking for perigraft gas/fluid collections, soft-tissue attenuation, pseudoaneurysms, or fistulous formations. PET-CT is useful for low-grade infections, showing high avidity within the infected area. 1 2 3 4

There is no standard treatment and a tailored approach should always be offered. The ultimate goal is to remove the infected graft, but this is not always feasible due to general fitness or comorbidities.

These cases are best discussed on an MDT formed by vascular and cardiovascular surgeons, radiologists, and specialists of infectious diseases to find a procedure that combines the best morbidity, mortality, and durability. 1 2 3 4

If there is no immediate danger to the patient's life, conservative treatment can be attempted with antibiotics guided by sensitivity of the causative organism when available; surgery should be attempted first in young and fit patients and in those unlikely to resolve conservatively (i.e., extensive perigraft purulence, pseudoaneurysm, or suspected aortoenteric fistula). Surgical treatment consists of removal of the infected graft followed by revascularization techniques using synthetic, autologous, or cryopreserved allografts either for in situ reconstruction or extra-anatomical bypass techniques.

Long-term antibiotic therapy and monitoring of CRP are usually required. 1 2 3 4

Mortality after infected abdominal EVAR is still significant despite the treatment used: 27 to 37% after surgery, 50% after endovascular, and 63% with conservative management. 5 6

*Neisseria meningitidis* appears as gram-negative cocci, oriented in pairs. *Neisseria spp.* inhabit the nasopharynx as commensals and are transmitted by close exposure through aerosolization of respiratory droplets or direct contact with secretions with an incubation period between 1 to 14 days. 7

With appropriate host humoral immune response, the invasive disease is prevented. However, with suboptimal immunity, invasion and spread will occur. Meningococcus utilizes a spectrum of virulence factors to evade the host-immune responses: capsule, immunoglobulin-A proteases, transferrin binding proteins, and surface blebs containing lipopolysaccharide which functions as an endotoxin and promotes cascade of proinflammatory cytokines (tumor necrosis factor-α, interleukin [IL]-I, IL-6, and IL-8) that lead to endothelial damage, capillary leak, procoagulant state and microthrombi formation.

Meningococcal infection varies from nonlocalized febrile illness to meningitis and/or septicaemia. 8

We believe that our patient had the initial bacteremia with the flu-like symptoms and the graft infected by the presence of the meningococci in the blood stream. Antibiotics were effective to treat the systemic but not the graft infection.

Most likely, the SIRS during surgery was consequence of *Neisseria meningitidis* release into the blood stream during the EVAR and perigraft collection manipulation leading to the effects described earlier.

To conclude, we report a rare case of infrarenal EVAR infection by *Neisseria meningitidis* . Despite a well-planned surgery, the patient could not overcome the perioperative SIRS following the removal of the infected prosthesis and died in the early hours after the operation.
